# Topics, Delivery Modes, and Social-Epistemological Dimensions of Web-Based Information for Patients Undergoing Renal Transplant and Living Donors During the COVID-19 Pandemic: Content Analysis

**DOI:** 10.2196/22068

**Published:** 2020-10-08

**Authors:** Charlotte W van Klaveren, Peter G M de Jong, Renée A Hendriks, Franka Luk, Aiko P J de Vries, Paul J M van der Boog, Marlies E J Reinders

**Affiliations:** 1 Division of Nephrology and Transplant Center Department of Internal Medicine Leiden University Medical Center Leiden Netherlands; 2 Center for Innovation in Medical Education Leiden University Medical Center Leiden Netherlands; 3 Division of Nephrology and Transplantation Department of Internal Medicine Erasmus University Medical Center Rotterdam Netherlands

**Keywords:** web-based information, internet, websites, patient education, COVID-19, renal transplantation, eHealth, constructivism, social-epistemological dimensions, teaching modes, health communication

## Abstract

**Background:**

The COVID-19 pandemic has markedly affected renal transplant care. During this time of social distancing, limited in-person visits, and uncertainty, patients and donors are relying more than ever on telemedicine and web-based information. Several factors can influence patients’ understanding of web-based information, such as delivery modes (instruction, interaction, and assessment) and social-epistemological dimensions (choices in interactive knowledge building).

**Objective:**

The aim of this study was to systemically evaluate the content, delivery modes, and social-epistemological dimensions of web-based information on COVID-19 and renal transplantation at time of the pandemic.

**Methods:**

Multiple keyword combinations were used to retrieve websites on COVID-19 and renal transplantation using the search engines Google.com and Google.nl. From 14 different websites, 30 webpages were examined to determine their organizational sources, topics, delivery modes, and social-epistemological dimensions.

**Results:**

The variety of topics and delivery modes was limited. A total of 13 different delivery modes were encountered, of which 8 (62%) were instructional and 5 (38%) were interactional; no assessment delivery modes were observed. No website offered all available delivery modes. The majority of delivery modes (8/13, 62%) focused on individual and passive learning, whereas group learning and active construction of knowledge were rarely encountered.

**Conclusions:**

By taking interactive knowledge transfer into account, the educational quality of eHealth for transplant care could increase, especially in times of crisis when rapid knowledge transfer is needed.

## Introduction

The COVID-19 pandemic is affecting transplant activity worldwide [1-3]. In addition to its impact on donation and transplantation numbers, the pandemic has markedly affected outpatient transplant care [1,2]. Additionally, patients experience uncertainty and fear; therefore, they rely more heavily on information provision [4]. For patients undergoing renal transplant, this uncertainty includes the fear of contracting COVID-19, the postponement of transplant procedures, screening strategies for COVID-19, mental health problems, and the risk of using immunosuppressive therapy during the pandemic [1,2]. With current measures limiting in-person visits between patients and health care providers, it has become vitally important to use alternative means of communication to inform patients undergoing renal transplant and living donors. Telephone and video communication have been generally adopted as means to continue outpatient care during the crisis [5,6]. Web-based information is used in the provision of eHealth to meet patients’ information needs when they are facing uncertainty and fear [7].

Multiple eHealth interventions aimed at disease control and increasing knowledge were already available for renal patients before the outbreak [8]. The majority of patients with renal disease search the internet on a regular basis to obtain additional information on their disease and its treatment [9]. In general, patients’ health-related internet use is associated with increasing health literacy [10]. Health literacy can be best described as an individual’s well-considered health decisions and goal setting by accessing and understanding health-related information [11]. Patients’ health literacy is positively associated with their self-management [12], motivation [13], risk perception [14], participation in health decision making [15,16], etc. However, despite the high number of patients with renal disease who use the internet as an additional information source, health literacy among renal patients and transplant recipients is still limited. A systematic review showed that in the United Kingdom, 25% of patients with renal disease not on dialysis, 27% of patients with renal disease on dialysis, and 14% of transplant recipients have limited health literacy [17].

Several factors could influence patients’ health literacy, such as their understanding of web-based health information [18]. As knowledge transfer is an essential element in initiating behavioral change, the effective use of delivery modes could play a role in understanding information about COVID-19 and achieving this change [19]. Despite the increased provision of web-based information and eHealth interventions relating to COVID-19, it is not known how web-based information has been provided during the pandemic [20].

Information can be conveyed through instruction, interaction, and assessment [21]. Various delivery modes can be used for this, such as text messages, discussions, and quizzes. Some of these modes focus on the passive transfer of factual information to the receiver (called objectivistic modes), whereas others focus on knowledge construction, information processing, hands-on interaction with the content, and problem-solving (constructivist modes) [22,23]. Constructivist learning promotes more active processing and personalization of information compared to receiving it passively. This results in deeper understanding and embedding of newly acquired knowledge [24-26]. In addition to this so-called epistemological dimension, delivery modes can also be categorized socially as individual or group learning [27]. From the literature, it can be assumed that people who learn both individually and collaboratively can construct knowledge better than learners who only learn individually [28].

Web-based information was rapidly developed by health services, academic centers, and patient associations to meet people’s information needs related to COVID-19 and transplantation. However, if patients’ understanding is to be optimized, the educational quality of this web-based information should be taken into account. The aim of this study is to provide a systematic overview of the source organizations, topics discussed, available delivery modes, and corresponding social-epistemological dimensions of websites for patients undergoing renal transplant and living donors on the topic of COVID-19. The practical implications derived from this study can be used to increase the quality of web-based information for transplant care, especially in times of crisis when rapid transfer of knowledge is needed.

## Methods

Websites on renal transplantation and COVID-19 were systemically identified using the most frequently consulted search engines for two countries [29]: Google.com [30] (for the United States, in North America), and Google.nl [31] (for the Netherlands, in Europe). The researcher's internet settings were deleted to obtain the cleanest and most objective search results possible. Additionally, the researcher's Google accounts, prerendering, and location sharing were turned off. The location setting was changed manually to the United States to minimalize the influence of the researcher’s location (the Netherlands) when using the search engine Google.com. Websites were searched from a laptop in the Netherlands in the month of March 2020.

To obtain a clearer picture of the available web-based source organizations, delivery modes, and social epistemological dimensions, general search engine queries related to renal transplantation and COVID-19 were used to obtain relevant websites. Because the English keywords *renal transplantation* and *COVID-19* have multiple synonyms, and patients might use any combination of those, a non-research–based selection of 14 keyword combinations was used to identify potentially relevant websites in English containing information on both renal transplantation and COVID-19 (Multimedia Appendix 1, columns A and C). In the Dutch search, a total of 7 keyword combinations were used (Multimedia Appendix 1, columns B and C).

Previous studies have demonstrated that searchers rarely read beyond the first 10 search results [32]. Therefore, in this study, websites were included if they were in the top 10 search results, including websites that used paid advertisements to top the search results. Front page news and suggested YouTube videos were not included. All possible English and Dutch keyword combinations were entered respectively in both Google search engines. The first 10 recovered websites for each search were included for detailed examination.

In this study, we were interested in publicly accessible and available websites on the World Wide Web that patients and donors could access on an ad hoc basis. Therefore, for each search, websites were excluded if the website was no longer available, the website was under construction, or the website’s content was behind a paywall. Websites were also excluded if the selected webpage was redundant (eg, press releases, news articles, blogs, academic journals, or webpages to sell products), or the webpage did not provide information on COVID-19 and renal transplantation. These websites were excluded because we were interested in websites which had as goal to objectively inform patients or living donors. Additional exclusion criteria were the website was a duplicate with a previous hit or the written language was other than English or Dutch, depending on the language of the keyword combination.

Potentially relevant webpages were obtained by using the same keyword combination in the website search function that was used for the website inclusion. The exclusion criteria for webpage selection per website were identical to those described for the website selection. Duplicated webpages obtained with different combinations of keywords were all noted. During data analysis, overlapping webpages were included only once. A total of 30 webpages, 15 in English and 15 in Dutch, were included for detailed examination.

Three randomly selected websites were individually examined by two authors. The results of both authors were discussed and calibrated until agreement on coding was reached. Then, the first author re-examined these three websites and examined the remaining websites. A calibration diary was maintained during examination, and another author was consulted when uncertainty arose.

Data analysis was performed in the month of April 2020. All websites were classified based on source organization, namely professional nonprofit organizations, such as hospitals; support groups, such as patient associations; governments, such as the Ministry of Health; individual practice, such as personal websites; or commercial organizations, such as independent dieticians. The website was labelled as “other” if the organizational source of a website did not match any of these categories. Additionally, English language websites were classified based on their generic top level domain (eg, .com, .org), which was not applicable to Dutch websites.

Each webpage was classified based on available topics and delivery modes. Immediately available content on COVID-19 and renal transplantation was categorized thematically. The available delivery modes on each webpage were classified into instruction, interaction, or assessment based on the studies of Toven-Lindsey et al (2015) [21] and Hendriks et al (2019) [33]. Delivery modes that were not predetermined were categorized individually by two authors, followed by discussion and calibration until agreement was reached.

After data collection was completed, the Teaching Approach Framework described in 2006 by Arbaugh and Benbunan-Fich [27] was used to categorize the identified delivery modes into social-epistemological dimensions: objectivist-individual; objectivist-group; constructivist-individual; and constructivist-group. Previously implemented categorizations by Toven-Lindsey et al (2015) [21] and Hendriks et al (2019) [33] were taken into account. However, in contrast to these studies, links to external web-based resources were categorized as objectivist-individual instead of constructivist-individual because external links available on websites transmit knowledge and do not actively build knowledge as designed for massive open online courses (MOOCs), which was the context of the prior studies. Newly found delivery modes were categorized into a social-epistemological dimension individually by two authors and discussed and calibrated until concurrence was reached.

Descriptive statistics were used to analyze the variety of organizational sources, content topics, delivery modes, and social-epistemological dimensions within and between websites.

## Results

### Organizational Sources of Websites

In total, 14 websites (7 English and 7 Dutch) were analyzed. The source organization of 8 of the 14 websites (57%) was a support group, 4 websites (29%) had a professional nonprofit organizational source, 1 website (7%) was for an individual practice, and 1 website (7%), WikiKids [34], did not match any of the given categories and was therefore labelled as “other.” All English websites had an organizational generic top level domain (.org).

### Topics Discussed on the Websites

**Figure 1:**
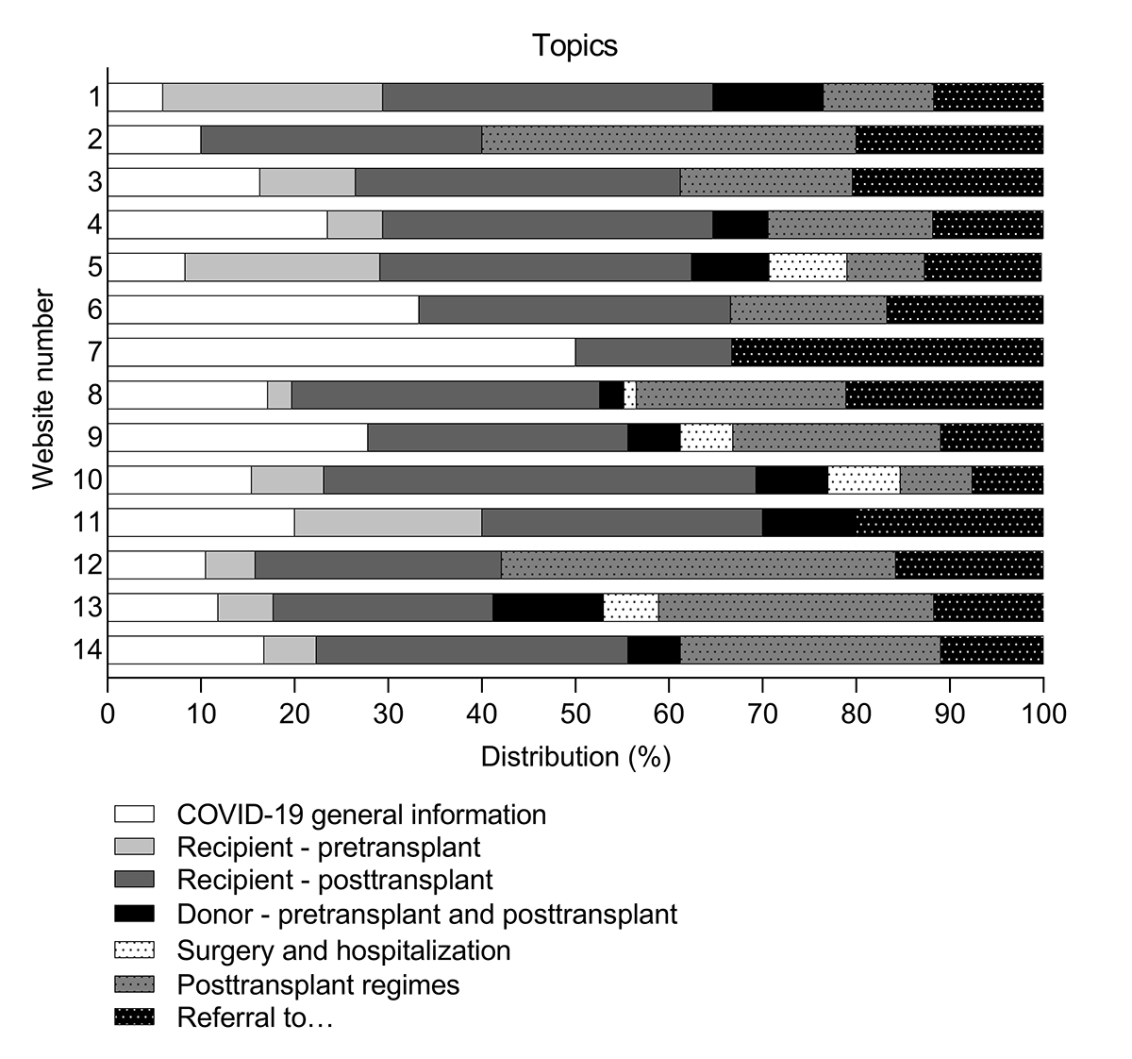
Distribution of different topics discussed on the included websites on COVID-19 for patients undergoing renal transplant and living donors, categorized in 7 main themes.

The topics discussed on all 30 included webpages were analyzed and covered by 7 main themes: COVID-19 general information, recipient–pretransplant, recipient–posttransplant, donor–pretransplant and posttransplant, surgery and hospitalization, posttransplant regimens, and referral to.... Of these 7 main themes, 3 (43%) were discussed on all 14 websites: COVID-19 general information, recipient–posttransplant, and referral to… (Figure 1). The minimum number of different main themes available per website was 3, and the maximum number was 7. A total of 4/14 websites (29%) discussed all main themes.

**Figure 2:**
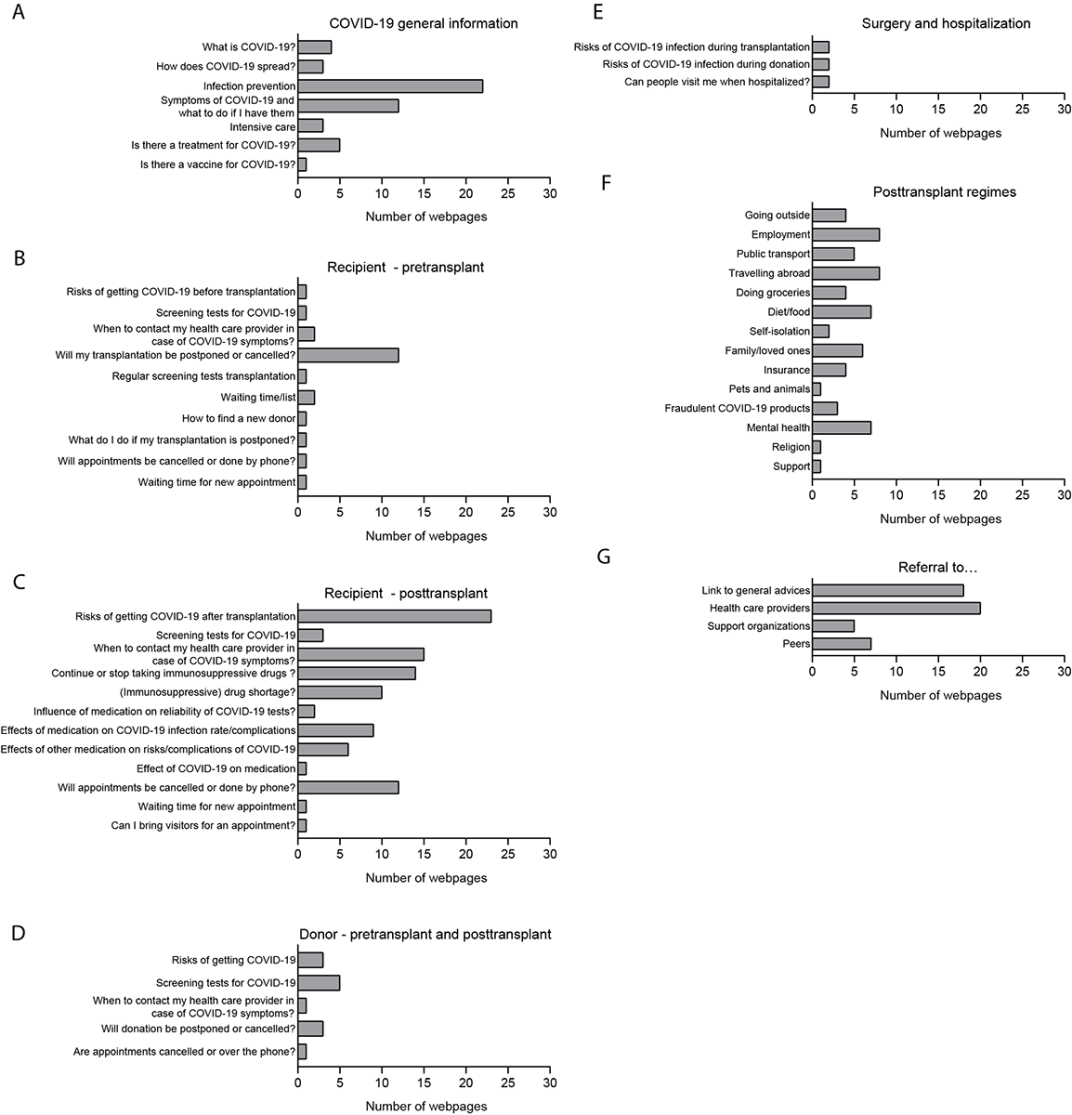
Numbers of webpages discussing content topics regarding COVID-19 for patients undergoing renal transplant and living donors. The webpages per included website that discussed the content topics were categorized in 7 main themes: (A) COVID-19 general information, (B) recipient–pretransplant, (C) recipient–posttransplant, (D) donor–pretransplant and posttransplant, (E) surgery and hospitalization, (F) posttransplant regimens, and (G) referral to….

Within the 7 main themes, a total of 56 different topics were discussed (Figure 2). Most topics were related to issues on posttransplant care for recipients, such as risks of contracting COVID-19 after transplant (23/30 webpages, 77%); when to contact health care providers in case of COVID-19 symptoms after transplant (15/30 webpages, 50%); and whether renal transplant recipients should continue or cease taking immunosuppressive therapy (14/30 webpages, 47%). In total, 14 different topics related to regimens were found on the 30 webpages, such as employment (8 webpages, 27%), travelling abroad (8 webpages, 27%), mental health (7 webpages, 23%), and diet (7 webpages, 23%). General information on COVID-19 infection prevention was discussed on 22/30 webpages (73%). Referrals to health care providers were encountered on 20 of the 30 webpages (67%), and 18 webpages (60%) offered links to general advice. Topics related to surgery and hospitalization and living donor information were found on 6/30 webpages (20%) and 13/30 webpages (43%), respectively.

### Delivery Modes on the Websites

**Figure 3:**
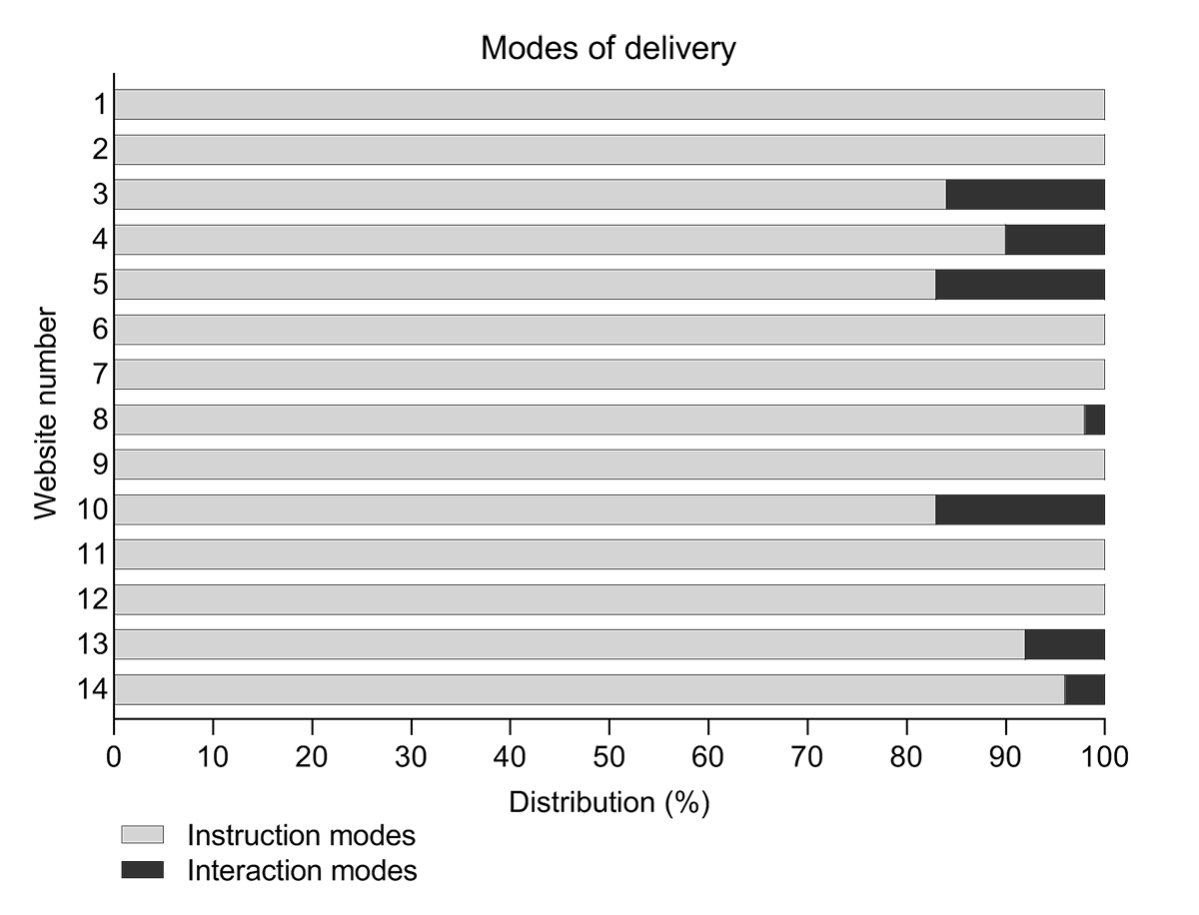
Distribution of delivery modes on each website.

A total of 13 different delivery modes were encountered (Table 1). Of 13 these modes, 8 (62%) were instructional, mainly links to external web-based resources and text. Of these 8 instruction modes, 3 (38%) were not predetermined by Toven-Lindsey et al [21] and Hendriks et al [33]: text-to-speech function, instruction video, and documentary. Of the 5 different interaction modes found, 4 (80%) were not predetermined: question submission form, survey, webinar, and one-on-one chat. Webinars and discussion boards for dialogue were the most commonly offered modes (5 times each). All examined websites offered instructional modes, and 7/14 websites (50%) offered interaction modes (Figure 3). Assessment modes were not observed on any of the included websites.

**Table 1:** Numbers of available delivery modes on the 14 included websites with a total of 30 webpages (N=270), n (%). No assessment modes were encountered.

Delivery mode		Value
**Instruction modes**		
	Text	30 (11.1)
	Link to external web-based resource	210 (77.8)
	Video of instructor talking to camera	1 (0.4)
	Illustration or simulation	9 (3.3)
	Digital textbook	2 (0.7)
	Documentary	1 (0.4)
	Instruction video	2 (0.7)
	Text-to-speech	1 (0.4)
**Interaction modes**		
	Discussion board for dialogue	5 (1.9)
	One-on-one chat	2 (0.7)
	Question submission form	1 (0.4)
	Survey	1 (0.4)
	Webinar	5 (1.9)

The different delivery modes (n=13) were offered 270 times in total, consisting mainly of links to external resources (210, 77.8%) and text (30, 11.1%) (Table 1). Limited variation of available delivery modes was observed between all websites: 256/270 (94.8%) of all available modes were instructional. Text was the only delivery mode that was encountered on all websites (Table 2). Both instruction and interaction modes were offered by 7 of the 14 websites (50%). However, none of the websites offered all available delivery modes. The minimum number of different modes available per website was 1 (text only), and the maximum number was 7.

**Table 2:** Presence of delivery modes per included website per search engine, classified as instruction or interaction mode.

Search engine		Google.nl							Google.com						
Website number		1	2	3	4	5	6	7	8	9	10	11	12	13	14
Webpages, n		1	1	7	1	3	1	1	8	1	1	2	1	1	1
**Instruction modes, n**															
	Text	1	1	7	1	3	1	1	8	1	1	2	1	1	1
	Link to external web-based resources	4	1	29	7	2	—^a^	35	73	7	4	4	18	11	15
	Video of instructor talking to camera	—	—	—	1	—	—	—	—	—	—	—	—	—	—
	Illustration or simulation	—	—	—	—	—	—	2	—	3	—	—	—	—	4
	Digital textbook	—	—	—	—	—	—	—	—	—	—	—	—	—	2
	Documentary	—	—	1	—	—	—	—	—	—	—	—	—	—	—
	Instruction video	—	—	1	—	—	—	—	1	—	—	—	—	—	—
	Text-to-speech	—	1	—	—	—	—	—	—	—	—	—	—	—	—
**Interaction modes**															
	Discussion board for dialogue	—	—	5	—	—	—	—	—	—	—	—	—	—	—
	One-on-one chat	—	—	—	—	—	—	—	2	—	—	—	—	—	—
	Question submission form	—	—	—	—	—	—	—	—	—	—	—	—	—	1
	Survey	—	—	1	—	—	—	—	—	—	—	—	—	—	—
	Webinar	—	—	1	1	1	—	—	—	—	1	—	—	1	—

^a^—: not applicable.

### Social-Epistemological Dimensions

**Figure 4:**
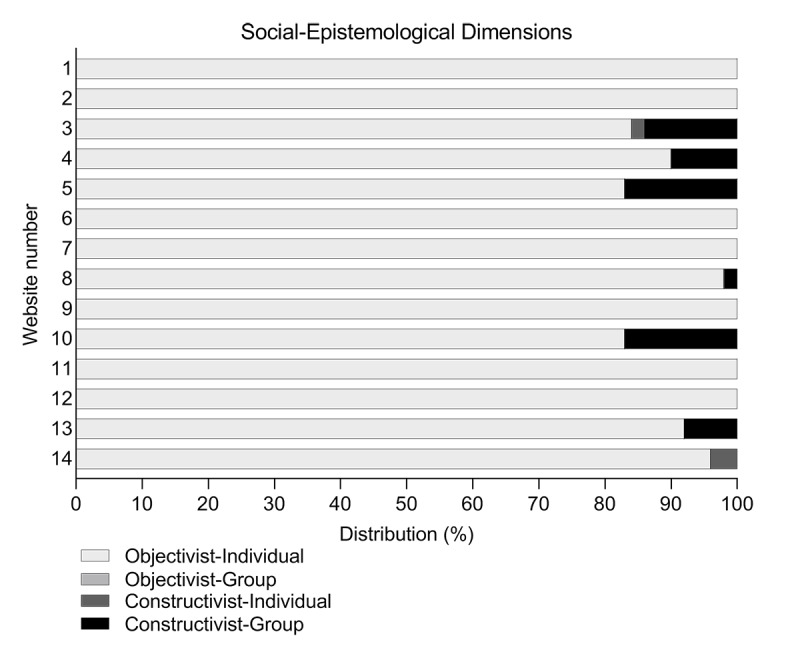
Distribution of social-epistemological dimensions per website.

In addition to the previously categorized delivery modes, the 7 nonpredetermined modes were classified into social-epistemological dimensions (Table 3) [21,33]. Text-to-speech functions, instruction videos, and documentaries were classified as objectivist-individual, whereas question submission forms and surveys were categorized as constructivist-individual. Webinars and one-on-one chats were categorized as constructivist-group. Of the 13 different delivery modes available, 8 (62%) were objectivist-individual, 2 (15%) were constructivist-individual, and 3 (23%) were constructivist-group. None of the offered delivery modes were within the objectivist-group dimension. The websites did not vary in the most commonly observed social-epistemological dimension (Figure 4). All of the 14 examined websites included objectivist-individual delivery modes, whereas constructivist-individual and constructivist-group modes were only offered by 2 (14%) and 6 (43%) websites, respectively. Individual-oriented delivery modes were observed the most frequently, with a minimum of 83% and a maximum of 100% per website.

**Table 3:** Social-epistemological dimensions of the delivery modes based on analysis of the social-epistemological dimensions according to the Teaching Approach Framework of Arbaugh and Benbunan-Fich [27] (N=270), n (%).

Delivery mode	Objectivist-individual	Objectivist-group	Constructivist-individual	Constructivist-group
Text	30 (11.1)	—^a^	—	—
Link to external web-based resource	210 (77.8)	—	—	—
Video of instructor talking to camera	1 (0.4)	—	—	—
Illustration or simulation	9 (1.9)	—	—	—
Digital textbook	2 (0.7)	—	—	—
Documentary	1 (0.4)	—	—	—
Instruction video	2 (0.7)	—	—	—
Text-to-speech	1 (0.4)	—	—	—
Discussion board for dialogue	—	—	—	5 (1.9)
One-on-one chat	—	—	—	2 (0.7)
Question submission form	—	—	1 (0.4)	—
Survey	—	—	1 (0.4)	—
Webinar	—	—	—	5 (1.9)
Total	256 (94.8)	0 (0.0)	2 (0.7)	12 (4.4)

^a^—: not applicable.

## Discussion

Since the COVID-19 outbreak, patients undergoing renal transplant and living donors have relied more than ever on telecommunication and web-based information because of fear and uncertainty about COVID-19 and limited in-person visits between the patient and health care provider. The aim of this study was to draw lessons from the topics conveyed and delivery modes used in web-based sources on COVID-19 and renal transplantation. The results show that the variety of content topics, delivery modes, and social-epistemological dimensions was limited. Additionally, the majority of the identified delivery modes focused on objectivistic and individual learning.

In our study, only 3 of the 7 main themes were discussed on all the websites. Additionally, 18 of the 30 webpages (60%) referred users to external sources, and 20 (67%) referred them to health care providers for the latest information and general advice. A logical explanation is that information on COVID-19 rapidly becomes outdated because of the dynamics of the pandemic, and time and financial investments are required to provide the latest information. Regarding the content, the most frequently discussed topics were related to posttransplant care, such as risks of contracting COVID-19 after transplant and whether recipients should continue taking immunosuppressive therapy. The variety of content topics and number of webpages for living donors were limited. Each of the content topics discussed for living donors was also found for transplant recipients. General information about infection prevention, symptoms of COVID-19, and suggestions of what to do if experiencing said symptoms were observed frequently. In addition, only a few webpages discussed issues relating to mental health, employment, insurance, and support. This is in concordance with a previous study that demonstrated that after transplant, the main focus of health care providers is often on dealing with the disease and treatment, whereas patients would also prefer information on managing life after transplantation, including social and emotional support [35].

Previous studies have already focused on the medical quality of web-based information about renal transplants [36,37]. They found that this information is often unvalidated, inaccurate, and unreliable. Here, we focused on information delivery modes because these may influence patients’ health literacy (eg, understanding medical information and indirectly promoting behavioral changes and coping strategies) [8,9,27,28]. We found a limited variety of delivery modes; the majority of these modes were instructional, mainly consisting of text and links to external resources. Additionally, we did not find any assessment modes (eg, quizzes) on the websites of interest. Literature shows that there is a need for more interactive patient education. However, the desired education modalities of patients with renal disease are currently unknown [38]. We would suggest that web-based sources should offer more assessment modes because these are crucial to evaluating knowledge and can assist patients by providing insight into their personal goals [21,39]. Moreover, assessing patients’ understanding is an important element of promoting prevention behavior [40].

The limited variety in delivery modes is in contrast to another web-based education platform, MOOCs, of which a greater variety can be found in the medical field. This is probably because there are fewer time constraints with MOOCs and a wide team of people are involved in their development, including education professionals [33,41]. MOOCs are not fully comparable to websites, as a MOOC is a course with a beginning and end and contains learning objectives to be achieved. However, taking the educational design of MOOCs into account when developing website content could help improve the knowledge or change the behavior of learners who access the websites. It is interesting to note that the London School of Hygiene & Tropical Medicine recently developed a MOOC for the general public, which focused on understanding and responding to COVID-19 by providing multiple modes: articles, videos, peer reviews, and quizzes [42].

Previous studies demonstrated that actively constructing information instead of passively transferring it results in better and deeper understanding and embedding of knowledge and behavioral changes [24-26]. The vast majority of the delivery modes in our study contained an objectivist-individual dimension and almost no applied constructivist learning. As renal patients must cope with new lifestyle regimens and in-person contact between patients and health care providers is often replaced by telemedicine, patients are expected to take a more active role. This includes monitoring their blood pressure and weight at home. To maintain these behavioral changes, a shift to more constructivist modes of information delivery is recommended.

Moreover, the integration of group learning, such as interaction with peers and participation in group activities, is favorable to learning [27]. Previous studies showed that group-based education for patients with type 2 diabetes promotes disease-specific knowledge, self-empowerment, and drug adherence, and it even improves clinical outcomes compared to individual education [43]. In addition to its effectiveness, patients favor group learning because it enables them to immediately receive answers to questions, discuss experiences and questions with peers, and experience a feeling of community [44]. To incorporate group learning during times of social distancing and limited in-person visits, a webinar may be a good option because this delivery mode offers the possibility of synchronous web-based interactive conversation between patients, living donors, and health care providers [45]. Additionally, contact with peers can increase patients’ self-management, and group education settings can help patients to overcome feelings of isolation [46]. Moreover, implementing webinars in transplant care could help health care providers tailor information to patients’ information needs because patients and living donors can submit questions beforehand.

A limitation of our study is that we mapped the available delivery modes on the included webpages at a single time point in April 2020. However, the variety of delivery modes may change over time. Therefore, future studies should analyze available delivery modes at multiple time points to investigate the dynamics and compare differences.

In conclusion, the variety of topics and delivery modes of web-based information on COVID-19 for patients who undergo renal transplants and living donors is limited. We therefore recommend providing information on COVID-19 in more diverse and interactive ways. Additionally, web-based sources should focus more on knowledge construction than on passive information transfer, and they should take interactivity into account. This is particularly important in times of crisis, when rapid knowledge transfer is needed.

## Appendix

Multimedia Appendix 1 Keyword combinations used to search for websites on renal transplantation and COVID-19 using the search engines Google.com and Google.nl.

## Abbreviations

MOOC: massive open online course
