# Supplementary figures and images for: Topics, Delivery Modes, and Social-Epistemological Dimensions of Web-Based Information for Patients Undergoing Renal Transplant and Living Donors During the COVID-19 Pandemic: Content Analysis
Source: J Med Internet Res. 2020 Oct 8;22(10):e22068. doi: 10.2196/22068 (PMC7546867; doi:10.2196/22068)

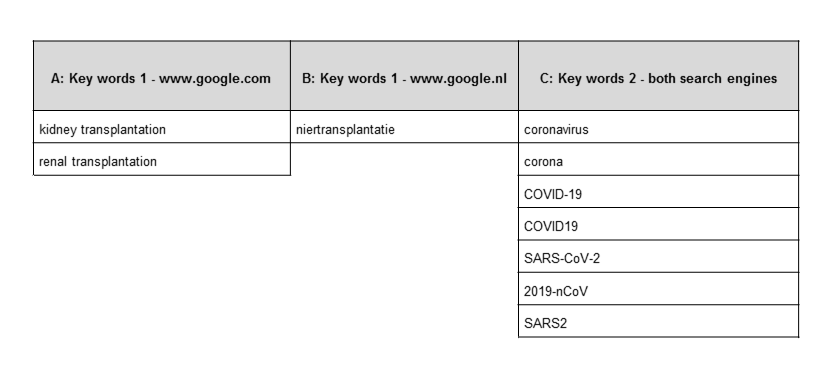

Supplement: Multimedia Appendix 1 [file jmir_v22i10e22068_app1.png]
